# Regenerative Effects of Locally or Intra-Arterially Administered BMSCs on the Thin Endometrium

**DOI:** 10.3389/fbioe.2022.735465

**Published:** 2022-04-25

**Authors:** Qi Guo, Yajie Chang, Jingjie Li, Chuanchuan Zhou, Rui Huang, Xing Yang, Guihua Liu, Xiaoyan Liang

**Affiliations:** Center of Reproductive Medicine, The Sixth Affiliated Hospital, Sun Yat-sen University, Guangzhou, China

**Keywords:** stem cells, thin endometrium, regenerative, angiogenesis, fibrosis

## Abstract

Stem cell–based therapy plays a pivotal role in the regeneration of damaged endometrium. Previous studies have demonstrated the therapeutic potential of bone marrow mesenchymal stem cells (BMSCs) through diverse administration ways. However, the homing, survival, and differentiation potential of these differently administered BMSCs are poorly defined, and the best route of administration is not well-defined. Herein, we aim to compare the engraftment, retaining time, and therapeutic efficiency of differently administered BMSCs. To achieve this, GFP/Luc-labeled BMSCs administered in two modes were assessed in a thin endometrium rat model: either into the damaged horns directly or through the ipsilateral iliac artery. The retaining time and hemi-quantitative distribution were evaluated by *in vivo* bioluminescence imaging and immunohistological analysis. Locally administered BMSCs were strongly detected in the abdomen at the first 4 days post treatment but underwent a rapid decrease in luminescent signal afterward and were rarely found 28 days after treatment. In contrast, the retaining time of BMSCs injected through the iliac artery was longer, reflected by more GFP-positive cells detected in the uterine section 28 days post treatment. Differentiation toward endometrial stromal cells was observed. Both routes of administration contributed to the restoration of the damaged endometrium, showing a comparable increase in the endometrial thickness and a decrease in fibrosis. However, more importantly, higher expression of LIF and VEGF, better recruitment, and longer retainment were found in the intra-arterial administration, contributing to the establishment of the optimal administration mode in clinical practice.

## Introduction

The optimal endometrial thickness for embryo implantation is still an open issue. Thin endometrium, defined as the thickness less than 7 mm on the day of human chorionic gonadotropin (hCG) administration, is identified to be related to low implantation rates and early abortion rates (Zhang et al., 2019; Shalom-Paz et al., 2021). Various treatments have been applied to increase endometrial thickness, including hormone replacement therapy, cytokine therapy, and hysteroscopic surgery, all of which show limited efficacy on the restoration of a functional endometrium (Kamath et al., 2020). Most of the thin endometrium is caused by repeated uterine curettage–led injury and chronic inflammation of the endometrium. The functional impairment or mechanical damage is responsible for decreased endometrial regenerative activities and increased implantation failure. The loss or senescence of uterine basal layer cells might impede endometrial reconstruction (Santamaria et al., 2018; Patterson et al., 2018).

Currently, cell-based therapy has emerged as a promising strategy in regenerative medicine. Owing to the easy isolation from various tissues and the properties of self-renewal, multipotency, and immunomodulation, MSC-based therapy has become a promising method for the thin endometrium. Mesenchymal stem cells (MSCs) have been used to treat the thin endometrium of patients with Asherman syndrome and improve reproductive outcomes (Azizi et al., 2018; Gao et al., 2019; Keyhanvar et al., 2021). Varied delivery ways were used in MSC perfusion. Cao et al. (2018) reported that the delivery of MSCs to the uterine cavity improved the endometrial thickness and reproductive outcomes. To better compensate for the insufficient intrinsic regeneration ability, collagen scaffolds were also used in the treatment. Santamaria et al. (2016)transplanted CD133^+^ BMSCs by the uterine artery to induce endometrial proliferation by engrafting around endometrial vessels of the traumatized endometrium. However, there is still no study focusing on comparing the effects of local perfusion or intra-arterially administered BMSCs on the regeneration of the thin endometrium. Therefore, we labeled BMSCs with both GFP and luciferin to track administered cells *in vitro* and *in vivo* by immunofluorescent analysis and bioluminescence imaging aiming to compare the distribution, survival, differentiation, and therapeutic efficacy of BMSCs administered either locally or intra-arterially.

## Materials and Methods

### Isolation and Culture of Primary Rat BMSCs

The isolation and culture of BMSCs were performed as previously described with some modifications. Briefly, the female Sprague–Dawley (SD) rats (3–4 weeks old, 80–100 g) were euthanized by CO_2_ asphyxiation. The marrow was flushed out from tibias and femora, and the isolated cells were cultured in low glucose Dulbecco’s modified Eagle’s medium (L-DMEM) supplemented with 10% fetal bovine serum, penicillin/streptomycin, and 4 ng/ml bFGF. The round cells began to adhere within 24 h after incubation, and the nonadherent cells were removed by changing the medium 48 h after incubation. The cells were then trypsinized and sub-cultured when reaching 80–90% of confluence. The cells between passages 3 and 6 were used in the following experiments.

### Assessments of the Immunophenotype and *in vitro* Differentiation Capability of BMSCs

The BMSCs of the third passage were used to detect the surface antigen markers. Adherent cells were harvested and resuspended in PBS and then incubated with FITC-labeled anti-CD90 (0.06 µg/test, Invitrogen, 11-0900-81) and PE-labeled anti-CD45 antibodies (0.25 µg/test, Invitrogen, 12-0461-82) in the dark at 4°C for 30 min. Unstained cells and isotype controls (0.06 µg/test, Invitrogen, 11-4724-81) served as a negative control. After staining, the expression of cell surface markers was analyzed by flow cytometry (Beckman).

To confirm the differentiation capability of isolated BMSCs, the third passage of cells was induced to differentiate into adipocytes, osteoblasts, and chondrocytes using specific induction media (RASMX-90031, RASMX-9004, RASMX-90021, Cyagen Biosciences Inc.). The BMSCs were seeded onto six-well culture plates at a density of 2×10^4^ cells/cm^2^ and cultured to 100% confluence. The culture medium was changed to adipocytic and osteogenic induction medium. After 21–28 days of induction, Oil Red O staining was carried out to detect lipid droplets, and Alizarin Red S staining was performed to detect the calcifying nodules.

### Transduction of BMSCs

BMSCs were transduced with a lentiviral vector expressing both GFP and firefly luciferase (Fluc) purchased from Jikai Co. (Shanghai, China). Then, 72 h after transduction, the transduction efficiency was evaluated by analyzing the GFP-positive cells under fluorescence microscopy, and the infected cells were sorted by flow cytometry sorting.

### Establishment of a Thin Endometrium Model and Administration of BMSCs

**FIGURE 1:**
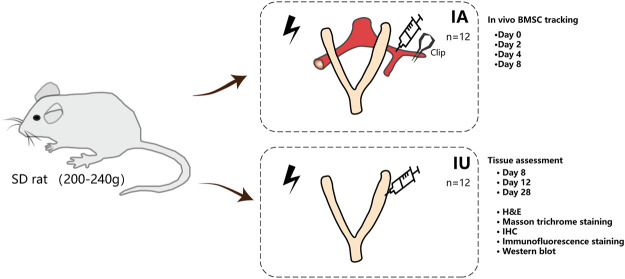
Study design and experimental schematic. BMSCs were isolated from the femora of rats and were labeled with EGFP and luciferase. BMSCs were injected into thin endometrium models either through the damaged horns directly or through the ipsilateral iliac artery when the external iliac artery was clamped by nontraumatic artery clips for less than 15 min. The rats were imaged by *in vivo* imaging systems immediately after closing the abdomen to confirm ideal infusion. At 2, 4, and 8 days post treatment, BMSC recruitment was evaluated by IVIS. The uteri were excised 8, 12, and 28 days after therapy and subjected to H and E, Masson’s trichrome, IHC staining, and Western blotting. For each group and each time point of examination, a size of three animals was used.

The animal study was reviewed and approved by the Animal Ethics Committee of the Sixth Affiliated Hospital, Sun Yat-sen University (No.20190307-002). The female SD rats were purchased from Beijing Vital River Laboratory Animal Technology Co. and bred at a temperature of 23–25°C with a 12 h light/dark cycle. After 1-week adaptive feeding, vaginal smears of the rats were performed to determine the estrous cycle, and only those with normal estrous cycles were chosen for subsequent experiments. A total of 48 rats were randomly assigned to four groups: normal, injured, intra-arterial, and intrauterine. The surgical procedure was performed under isoflurane anesthesia. For all rats, except for those in the normal group, the abdomen was open, and both sides of the uterus were carefully picked out to inject 500 μl of 95% ethanol in a sterile condition. The ethanol was aspirated out after 5 min; then, the uterine cavity was rinsed with physiological saline twice. For the intra-arterial and intrauterine groups, 1×10^6^ GFP/Luc-labeled BMSCs resuspended in 100 and 20 μl of PBS, respectively, were transplanted unilaterally after modeling. The injured group did not undergo any therapy. For each group and each time point of examination, a size corresponding to three animals was used (Figure 1).

### *In Vivo* Tracking of BMSCs

To detect the distribution of BMSCs comprehensively, the rats were imaged continuously by PerkinElmer IVIS Spectrum at 1, 2, 4, and 8 days after BMSC treatment. Briefly, the rats received a single intraperitoneal injection of 150 mg/kg firefly D-luciferin potassium salt (MB1834, Meilun Biotechnology Co. LTD.). The photographic and luminescent images were recorded by the camera 15 min after injection. For further comparison, the luminescent signal was recorded as total flux (average photos per second, p/s), and the region of interest (ROI) of constant size and position among different individuals was manually selected to quantify the luminescent intensity.

### *In Vitro* Assessment of GFP-Positive BMSCs

To further determine the precise location, quantity, and differentiation of administered BMSCs, immunofluorescence analysis was performed. The paraffin-embedded uteri slides were dewaxed in xylene and rehydrated through graded ethanol concentrations; then, antigen retrieval was performed by the microwave method in citrate solution. The uteri sections were incubated with anti-GFP mouse monoclonal antibody overnight at 4°C, followed by cy3-conjugated goat anti-mouse IgG secondary antibody (1:500, E031610-01, EarthOx) for 1 h in the dark, and stained with DAPI (meilunbio) at 10 μg/ml for 5 min. After that, the sections were examined under a laser scanning confocal microscope (Leica SP8). For differentiation detection, double staining was carried out. The sections were incubated with anti-GFP mouse antibody and anti-vimentin rabbit antibody (1:200, ABCAM, ab52942).

### Histological Assessment of the Endometrium Treated With Differently Administered BMSCs

Hematoxylin–eosin staining (H&E) was used to analyze the tissue structure. The uteri were fixed in 4% paraformaldehyde (PFA) solution overnight and embedded in paraffin, cut into 5-μm sections, and then stained with H&E.

To examine the collagen deposition in the injured endometrium, Masson’s trichrome staining was carried out. The extent of fibrosis among different groups, defined as the areas occupied by collagen fiber relative in proportion to the entire endometrium, was quantified by the software Image Pro Plus 6.0.

For immunohistochemistry staining (IHC), the slides were dewaxed in xylene and rehydrated through graded ethanol concentrations. Antigen retrieval was performed in citrate solution, and endogenous peroxidase activity was blocked in 3% H_2_0_2_ for another 15 min. The sections were incubated with anti-cytokeratin (1:200, Abcam, ab7763), anti-vimentin (1:200, Abcam, ab52942), anti-VEGF (1:200, Abcam, ab1316), and anti-ki67 (1:200, Abcam, ab16667) antibodies overnight at 4°C, followed by goat anti-mouse IgG or goat anti-rabbit IgG secondary antibody for 60 min, and then stained with DAB substrate kit (ZSGB-BIO, China) and hematoxylin in accordance with the manufacturer’s protocol.

### Western Blotting

The expression of cytokeratin, vimentin, VEGF, LIF, and integrin β3 was analyzed by Western blotting. Briefly, equal amounts of proteins extracted from different groups were separated by 10% SDS-PAGE electrophoresis and transferred to PVDF membranes. The membranes were incubated with anti-cytokeratin (1:1,000 Abcam, ab7763), anti-vimentin (1:1,000, Abcam, ab52942), anti-VEGF (1:1,000, Abcam, ab1316), anti-TGF-β1 (1:1,000, Abcam, ab215715), and anti-ki67 (1:1,000, Abcam, ab16667) antibodies overnight at 4°C after blocking in 5% BSA and then incubated with HRP-conjugated goat anti-mouse or anti-rabbit secondary antibody at room temperature for 1 h on the second day. β-actin was used as a loading control.

### Statistical Analysis

All data were expressed as mean ± SD, and statistical analysis was performed by SPSS version 24.0. Student’s t-test or one-way ANOVA were applied to analyze the data. The differences were considered statistically significant when *p* < 0.05. GraphPad Prism 8.0 software was used to draw scientific graphs.

## Results

### BMSCs Possess Specific Immunophenotype and Multilineage Differentiation Capacity

**FIGURE 2:**
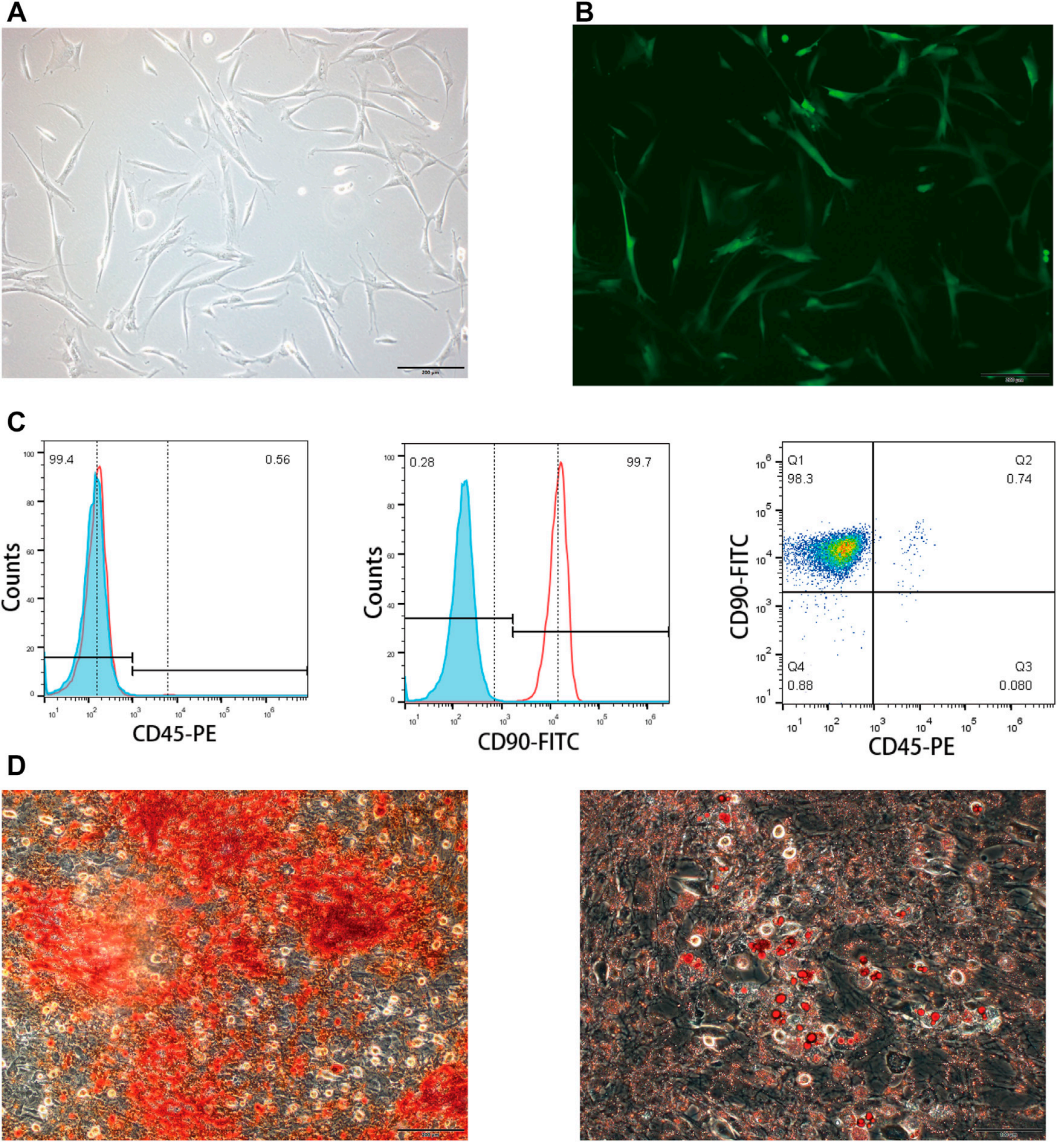
Characterization of rat BMSCs. **(A)** Bright field image showed the typical fibroblast morphology of purified BMSCs. **(B)** EGFP expression of transfected BMSCs measured by a fluorescence microscope. **(C)** Immunophenotype of BMSCs was examined by flow cytometry; CD90 was the positive marker and CD45 was the negative marker. **(D)** Oil Red O and Alizarin Red S staining were carried out to determine the differentiating abilities of BMSCs.

The isolated BMSCs displayed typical fibroblast morphology after purification (Figures 2A,B). Flow cytometry analysis was carried out to confirm the immunophenotype of BMSCs at passage 3. The expression of CD90 and CD45 was 99.7 and 0.56%, respectively (Figure 2C), showing specific immunophenotypes of BMSCs. In addition, BMSCs exhibited the capacity to differentiate into adipocytes and osteoblasts after induction (Figure 2D). Taken together, the BMSCs used in the following experiments match the MSC criteria.

The distribution of differently administered BMSCs by bioluminescence imaging and immunofluorescence analysis showed that the survival and retention time of BMSCs were longer in the intrauterine group.

**FIGURE 3:**
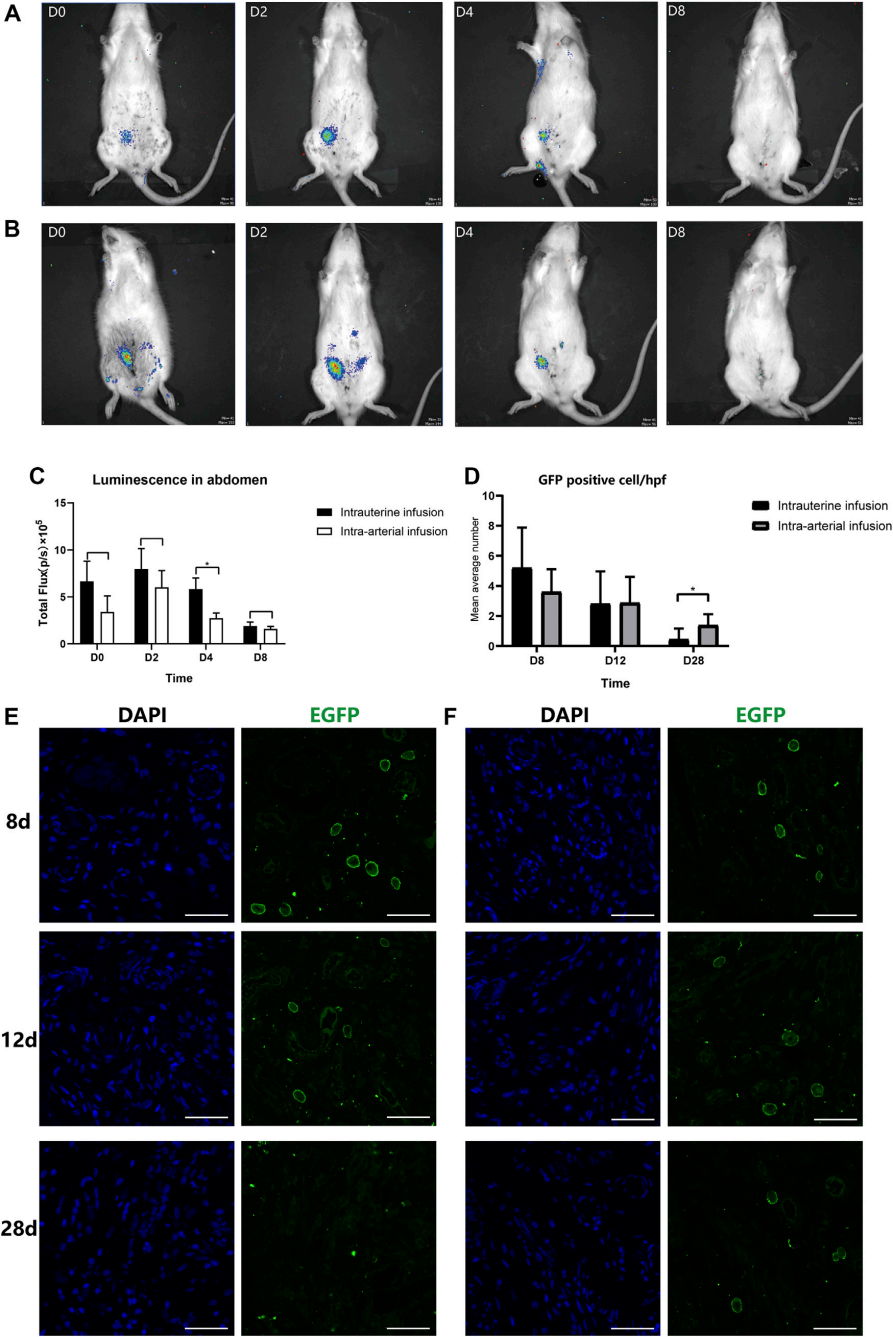
*In vivo* and *in vitro* tracking of transplanted BMSCs. The recruitment and retention of BMSCs at the injured horn in two groups (**(A)**: intra-artery, IA; **(B)**: intrauterine, IU) were monitored by IVIS at different time intervals (immediately following transplantation, 2, 4, and 8 days post transplantation); **(C)** Luminescence signals were quantified and recorded as total flux (p/s). Uteri were collected at 8, 12, and 28 days post treatment; a confocal microscope was used to track transplanted BMSCs in IU **(E)** and IA **(F)** groups after staining with anti-EGFP antibody and DAPI. **(D)** GFP-positive cells were counted under a confocal microscope by taking the mean value of three randomly selected high-power fields (hpf).

The BMSC recruitment was monitored at different time intervals (immediately following transplantation, 2, 4, and 8 days post transplantation) by an *in vivo* imaging system (Figures 3A,B). When BMSCs were infused into the lumen of the right horn directly, the luminescent signal remained distinct in the abdomen with no signals in other organs such as lungs in the first 4 days post treatment, indicating the precise and concentrated distribution of BMSCs when administered locally. However, in the subsequent 4 days, the signal underwent a rapid decrease and could barely be detected by bioluminescence imaging.

While infusing BMSCs through the ipsilateral iliac artery, the results were comparable. There were no significant differences in the uterine cavity between two different treating groups, and the signal was strong in the first 4 days and disappeared when tested at 8 days post treatment. However, the total fluorescence intensity was stronger following local administration (7.98×10^5^ and 6.02×10^5^ at day 2 and 5.83×10^5^ and 2.74×10^5^ at day 4. Figure 3C). It was assumed that a small number of cells entered the peripheral circulation when administered *via* the artery. Furthermore, we found that two individuals in the intra-artery group had signals in their right feet, and there were no signals detected in the abdomen (Supplementary Figure S3). We supposed that it was probably due to the anatomical differences in the diameter ratio of the internal iliac artery and external iliac artery, the major feeding artery of the lower limb. These individuals were excluded from subsequent analysis.

Since the signals were barely detected in either group after 8 days post treatment, the immunofluorescence analysis was then performed to further assess the survival and retention time of BMSCs in the two groups. As expected, the proportion of GFP-positive cells in the injured horns showed an overall similar declining trend from 8 to 28 days after therapy (Figure 3D). However, there was a significant decrease of rate in the intrauterine group, with an average of 5.67 and 4/HPF GFP-positive cells located in the injured endometrium at day 8 in the intrauterine and intra-arterial groups, respectively. At the 28th day post treatment, GFP staining remained low in the basalis layer of the endometrium in the intra-arterial group, and there were no GFP fluorescence signals that could be detected in the intrauterine group (Figures 3E,F).

**FIGURE 4:**
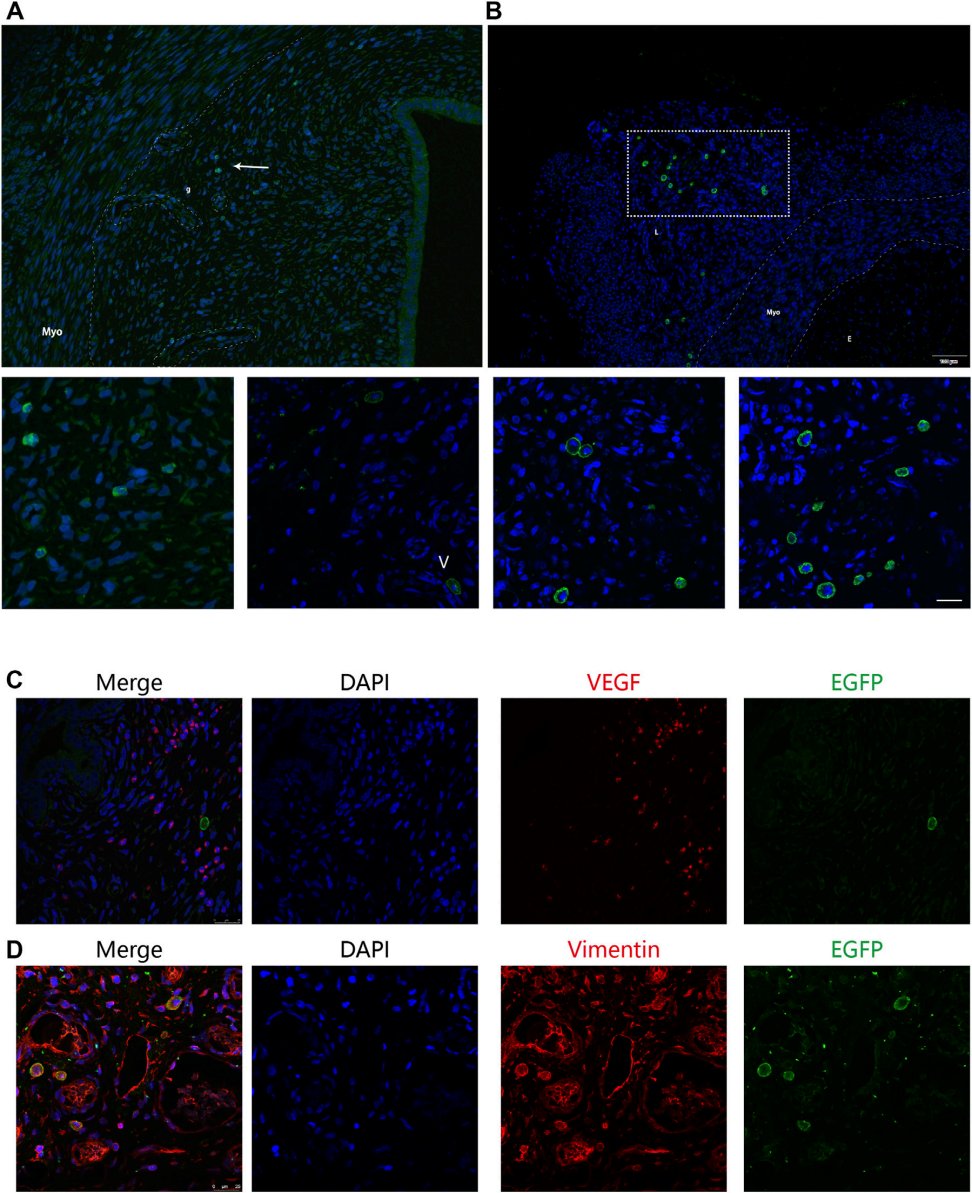
Precise distribution and differentiation of GFP + BMSCs. The murine uterus tissue consists of endometrial (glandular and luminal) epithelium, stroma, and myometrium. **(A)** Nearly all the GFP-positive cells were localized in the stroma, but not in the epithelium or myometrium. The distribution of GFP-positive cells is concentrated around the vessels and glands, indicated by the white arrow. g: glands, V: vessels. **(B)** Some GFP-positive cells were located around the vessels in the surrounding ligaments of uterus in the intra-arterial infusion group. Myo: myometrium; E: endometrium; L: surrounding ligaments. The uterine sections were stained with anti-GFP and anti-vimentin **(C)** or anti-VEGF **(D)** GFP^+^ BMSCs co-localized with vimentin marker but not VEGF.

We then compared the precise distribution and differentiation of the BMSCs delivered in two strategies by immunofluorescence analysis to explore the causes of difference in the BMSC retention time. The murine uterus tissue consists of endometrial (glandular and luminal) epithelium, stroma, and myometrium. We observed nearly all the GFP-positive cells localized in the stroma but not in the epithelium or myometrium. In addition, in terms of specific localization patterns, there was significantly more positive staining in the basal layer of the endometrium close to the glands and vessels than in the outer layer of the endometrium in the intra-arterial group (Figure 4A). Furthermore, in the intra-arterial infusion group, some GFP-positive cells were located around the vessels in the surrounding ligaments of the uterus (Figure 4B), confirming the feasible targeted delivery of BMSCs by our method.

To further explore the fate of transplanted cells, uterine sections from two groups at 12 days after therapy were double-stained by anti-GFP antibody and anti-vimentin or anti-VEGF antibody and examined under fluorescence microscopy, considering their specific distribution in the basal layer of the endometrium. Some of the stromal cells were both EGFP- and vimentin-positive, suggesting that MSCs were not only recruited but also began to differentiate into other cell types to restore the endometrium (Figures 4C,D). However, the proportion of recruited BMSCs was quite small. It is hard to capture GFP signals in the uterine sections at the 28th day post treatment, indicating that MSCs most likely do not exert their therapeutic effects through cellular replacement of the injured endometrium.

BMSCs administered differently were equally effective in reducing collagen deposition in different BMSC administered groups; VEGF and integrin β3 were higher expressed when transplanted through the artery.

**FIGURE 5:**
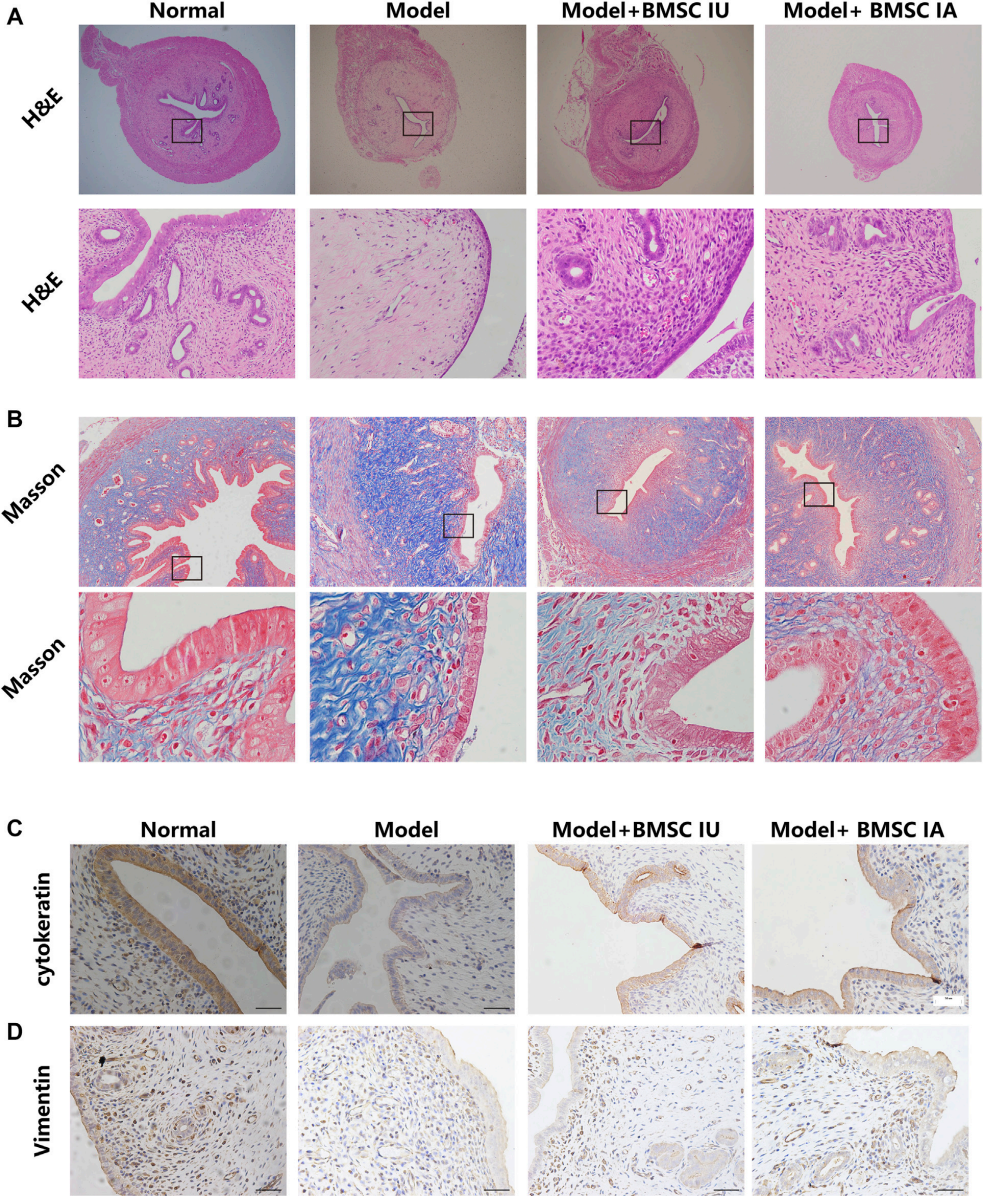
Morphological evaluation of the regenerative effect of BMSCs administered differently on the thin endometrium. The endometrium morphologies in the four groups (normal, model, BMSC IU-injection, and BMSC IA-injection) were examined by H&E staining **(A)** and Masson’s trichrome staining **(B)**. H&E indicates hematoxylin and eosin. Immunohistochemical analysis of the cytokeratin **(C)** and vimentin **(D)** expression in the four groups.

As shown in Figure 5, the modeling rats had a relatively thinner endometrium, and the intact structure was damaged with a remarkably decreased number of vessels and glands and increased interstitial edema. Some of the epithelial cell layers even became discontinuous, confirming the successful modeling as previously reported.

The H&E staining showed significantly increased endometrial thickness in both intra-arterial and intrauterine infusion groups compared to the model group, and the density of blood vessels and glands was also increased. However, in some individuals, the morphology of luminal epithelial cells remained flattened even at the 28th day after BMSC transplantation as compared with columnar epithelial cells in the normal control group, reflecting the incomplete endometrial repair by BMSCs. We then compared the morphological discrepancies of different groups at a consecutive time after BMSC treatment by H&E staining, Masson’s trichrome staining, immunohistological analysis, and Western blotting.

**FIGURE 6:**
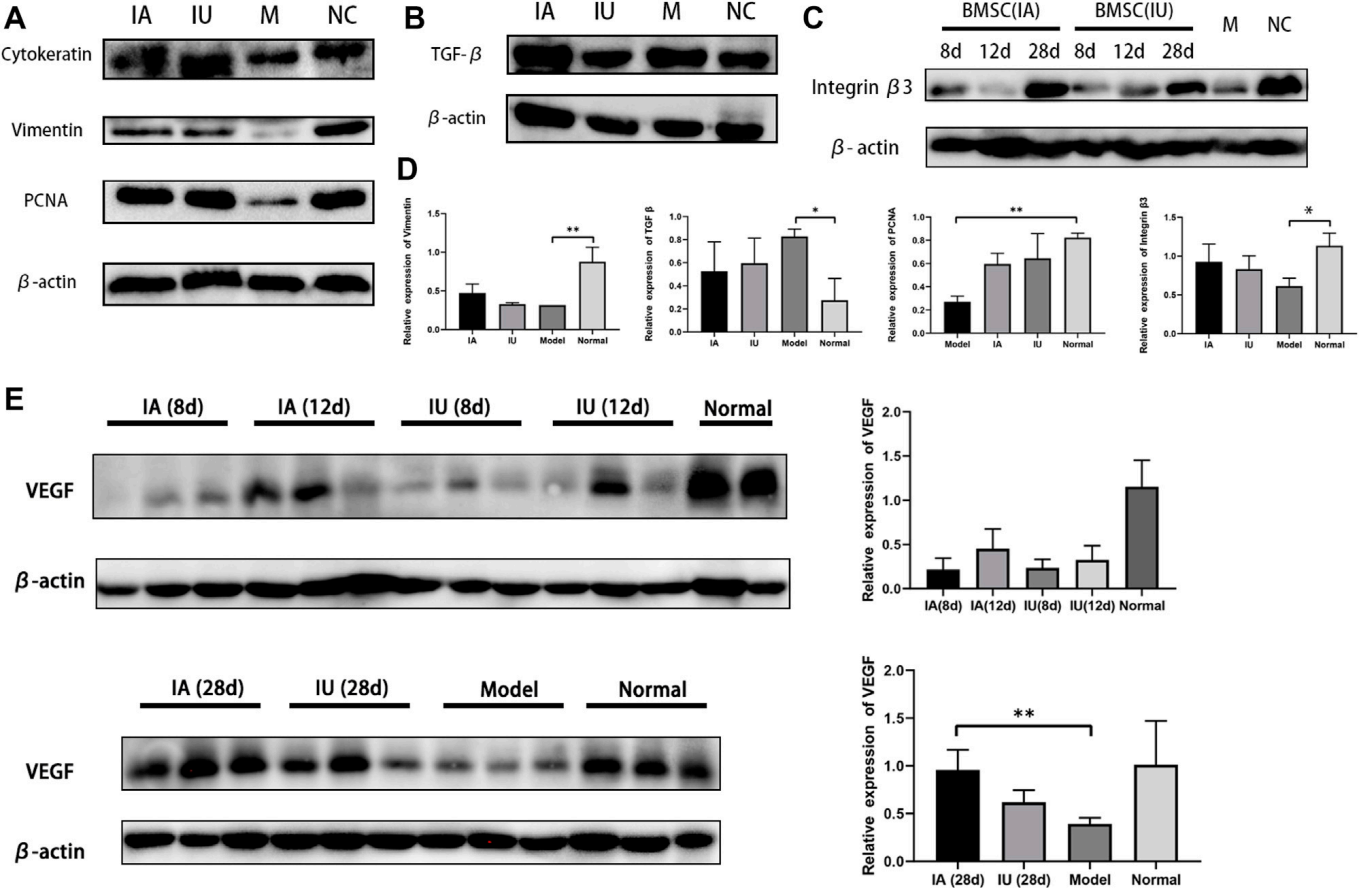
Representative Western blot results of cytokeratin, vimentin, PCNA, TGF-β1, and VEGF. **(A,B)** Cytokeratin, vimentin, PCNA and TGF-β1 showed a comparable increase in the two BMSC-infused groups (group IA and IU) **(C)** Differential expression of integrin β3 in four groups at the same time point. **(D)** The semi-quantification analysis of the expression of Vimentin, TGF-β1, PCNA, Integrin β3 in four groups, achieve by Image J software. **(E)** Expression of VEGF in four groups at different time points post modeling. The expression levels analyzed by ImageJ quantification are shown on the right. (NC, normal; M, model; IU, BMSC IU-injection; IA, BMSC IA-injection).

The results showed increased fibrosis after modeling and no obvious change in the collagen deposition in the 12 days post therapy in both groups, despite the mitigation of other injury indicators. At the 28th day post treatment, a comparable reduction in tissue fibrosis was observed in the two groups (Figure 5B). These histological results were consistent with the results of the Western blot, in which the expression of TGF-β1 only showed a slight change on the 28th day after therapy (Figure 6B). No obvious differences were observed when comparing these protein expressions in these two groups at consecutive time after therapy. Thus, we concluded that BMSCs administered differently were equally effective in reducing collagen deposition by regulating the TGF-β signaling pathway.

To further compare the therapeutic effects of differently administered BMSCs, we evaluated the expression of cytokeratin, vimentin, PCNA, LIF, integrin β3, and VEGF by immunohistochemical analysis and Western blotting. Cytokeratin and vimentin, the markers of epithelial and stromal cells, showed an increase in the two BMSC-infused groups (Figure 6A), and the protein expression of PCNA, the cell proliferation marker, increased significantly after BMSC infusion, which indicates the regeneration of both epithelial and stromal cells after BMSC therapy (Figure 6A).

Integrin β3 and LIF are the markers of endometrial receptivity and essential for successful embryo implantation. Our results demonstrated an increased expression of integrin β3, suggesting that BMSCs not only promoted endometrial renewal but also improved endometrial receptivity. Another interesting finding was the differential expression of VEGF and integrin β3 in the two groups at the same time point (Figures 6C,D). VEGF and integrin β3 were much higher in the intra-arterial infusion group at day 12 post treatment, implying that BMSCs promoted angiogenesis by upregulating related cytokines when transplanted through the ipsilateral iliac artery.

## Discussion

In this study, we compared the engraftment, retaining time, and therapeutic efficiency between locally or intra-arterially administered BMSCs. Intra-arterial and local administrations of BMSCs both contributed to the restoration of the damaged endometrium, showing a comparable increase in the endometrial thickness. However, trans-arterial BMSC therapy is more effective for patients with a severely thin endometrium.

Plenty of studies have shown the therapeutic potential of MSCs in the thin endometrium and Asherman’s syndrome models (Kilic, et al., 2014; Liu et al., 2019; Fan et al., 2021). One of the major obstacles in MSC therapy is the limited engrafted transplanted cell numbers and short retaining time. The therapeutic effects of MSCs from different sources or administration methods have been revealed by previous studies, whereas few studies compared the efficacies of different transplantation routes. Meanwhile, the current results are in controversy. Ouyang reported an equal therapeutic efficacy of MSCs in rat models of intrauterine adhesions, irrespective of transplantation routes (Monsef et al., 2020). Nevertheless, in the study by Liu et al. (2018), the proportion of GFP-positive BMSCs administered intravenously or locally engrafted to injured horns is 0.261 versus 0.045% at 2 weeks and 0.22 versus 0.058% at 3 weeks. Based on previous studies, it was conservatively concluded that MSCs administered intravenously were slightly superior or almost similar to those administered locally, despite a large part of cells being filtered out in the lungs (Santamaria, et al., 2018; Kallmeyer, et al., 2020). The intravenous administration might play roles in the endometrial tissue repair in an indirect manner, and we speculated that a smaller number of MSCs could possibly achieve the same regenerative effect when transplanted *via* vascular systems. Santamaria et al. (2016)found that CD133^+^ BMSCs transplanted *via* the uterine artery engrafted around endometrial vessels of the traumatized endometrium and induced endometrial proliferation. However, whether transplantation *via* artery results in better recruitment and longer retainment and finally enhances the therapeutic efficacy of transplanted cells is still unknown.

In our study, we compared the fate and regenerative effects of GFP/Luc-labeled BMSCs administered either through the ipsilateral iliac artery or locally using a thin endometrium rat model. The histological results showed that both the administration routes promote endometrial regeneration, with increased density of vessels and glands and decreased level of collagen deposition. We revealed that BMSCs administered either systematically or locally increased the expression of cytokeratin, vimentin, LIF, integrin β3, and VEGF and decreased the expression of TGF-β1. BMSCs promoted the reconstruction of the injured endometrium as previously reported. To further unravel the differences in therapeutic efficacy, several indicators such as cytokeratin, vimentin, VEGF, LIF, integrin β3, and PCNA were compared. Integrin β3 and VEGF showed a more obvious improvement in the intra-arterial infusion group, while other markers were comparable. VEGF is one of the most important factors facilitating angiogenesis; thus, we could preliminarily infer that BMSCs possess a higher pro-angiogenesis capacity when administered through intra-arterial infusion than intrauterine infusion. Although the underlying reasons could not yet be readily explained in our study, we believed that this pro-angiogenesis advantage of our approach over traditional methods is crucial since the vascularization deficiency is the major cause of the thin endometrium and appears to be the key obstacle in endometrial regeneration, leading to implantation failure (Alfer, et al., 2017; Han et al., 2020).

We found that a higher proportion of BMSCs recruited to the injured lumen was found in the intrauterine infusion group than in the intra-arterial infusion group in the first 4 days post transplantation. The number of GFP-positive cells retained in the injured endometrium in the intra-arterial infusion group was not less than that in the intrauterine infusion group at day 28 post treatment, and the ultimate therapeutic effects were comparable. Most of the cells disappeared soon after transplantation, and a rather low proportion of BMSCs were recruited and retained in the injured horns in both the groups, which was consistent with the previous reports that MSCs accounted for only 0.6% of the total uterine cells (Cervelló et al., 2015).

Some researchers presented evidence of human-derived MSCs differentiating into endometrial stromal cells in murine models (Zheng et al., 2020); we raised a question whether the immunogenicity of differentiated cells would increase. A similar differentiation of allogeneic BMSCs was observed in our study. Immunofluorescence analysis showed a fraction of GFP + cells co-localized with vimentin, which prompts us to consider the differentiation occurred during the therapy. However, even if the differentiation capability is proven to have occurred, these differentiated cells play a minor effect in the tissue repair due to their small percentage (Cervelló et al., 2015). In addition, due to the fact that the BMSCs leaking into peripheral circulation was inevitable, the initial BMSC engraftment in damaged horns was probably unequal in the two treating groups. Thus, it was speculated that a small number of BMSCs could achieve the same regenerative effects as the traditional delivering method.

The endometrium is a highly regenerative tissue. In a woman’s whole reproductive age, the endometrium undergoes over 400 cycles of proliferation and shedding. It was stated that endometrial stem cells and their microenvironment (niche) are responsible for the monthly endometrial renewal. It was reported that BMSCs were predominantly localized around blood vessels when transplanted through the tail vein. We, therefore, assumed that the transplanted BMSCs exerted therapeutic effects mostly *via* paracrine mechanisms by stimulating and improving the repair capacity of endometrial stem cells. However, due to the lack of reliable and specific markers for isolating or examining (Tempest et al., 2018), it is hard to track the changes of endometrial stem cells throughout the treatment. We found that more GFP-positive cells presented a perivascular location when infused through the ipsilateral iliac artery, and the percentage of GFP-positive cells located around the vessels increased with the extension of time, which indicated that the specific perivascular location could promote BMSC survival and probably their regenerative effects at the same time.

## Conclusion

In this study, we found that intra-arterial and local administrations of BMSCs both contributed to the restoration of the damaged endometrium, showing a comparable increase in the endometrial thickness. However, as the low viability and persistence of MSCs are the major obstacles of therapeutic efficacy, we suggest intra-arterial administration as a superior alternative to other delivering methods since it results in better recruitment and longer retention; meanwhile, it avoids pulmonary embolism. We also observed a higher pro-angiogenesis capacity in BMSCs administered arterially. Overall, trans-arterial BMSC therapy is more effective for patients with a severely thin endometrium.

## Data Availability

The original contributions presented in the study are included in the article/Supplementary Material, further inquiries can be directed to the corresponding authors.
