# Supplementary material for: Regenerative Effects of Locally or Intra-Arterially Administered BMSCs on the Thin Endometrium
Source: Front Bioeng Biotechnol. 2022 Apr 25;10:735465. doi: 10.3389/fbioe.2022.735465 (PMC9081369; doi:10.3389/fbioe.2022.735465)
Supplement: Supplementary file 1 [file Table1.DOCX]

Raw data is available online at the following link: <https://www.jianguoyun.com/p/DVdIJakQ-KzSCRiusYIE>
