# Supplementary figures and images for: Regenerative Effects of Locally or Intra-Arterially Administered BMSCs on the Thin Endometrium
Source: Front Bioeng Biotechnol. 2022 Apr 25;10:735465. doi: 10.3389/fbioe.2022.735465 (PMC9081369; doi:10.3389/fbioe.2022.735465)

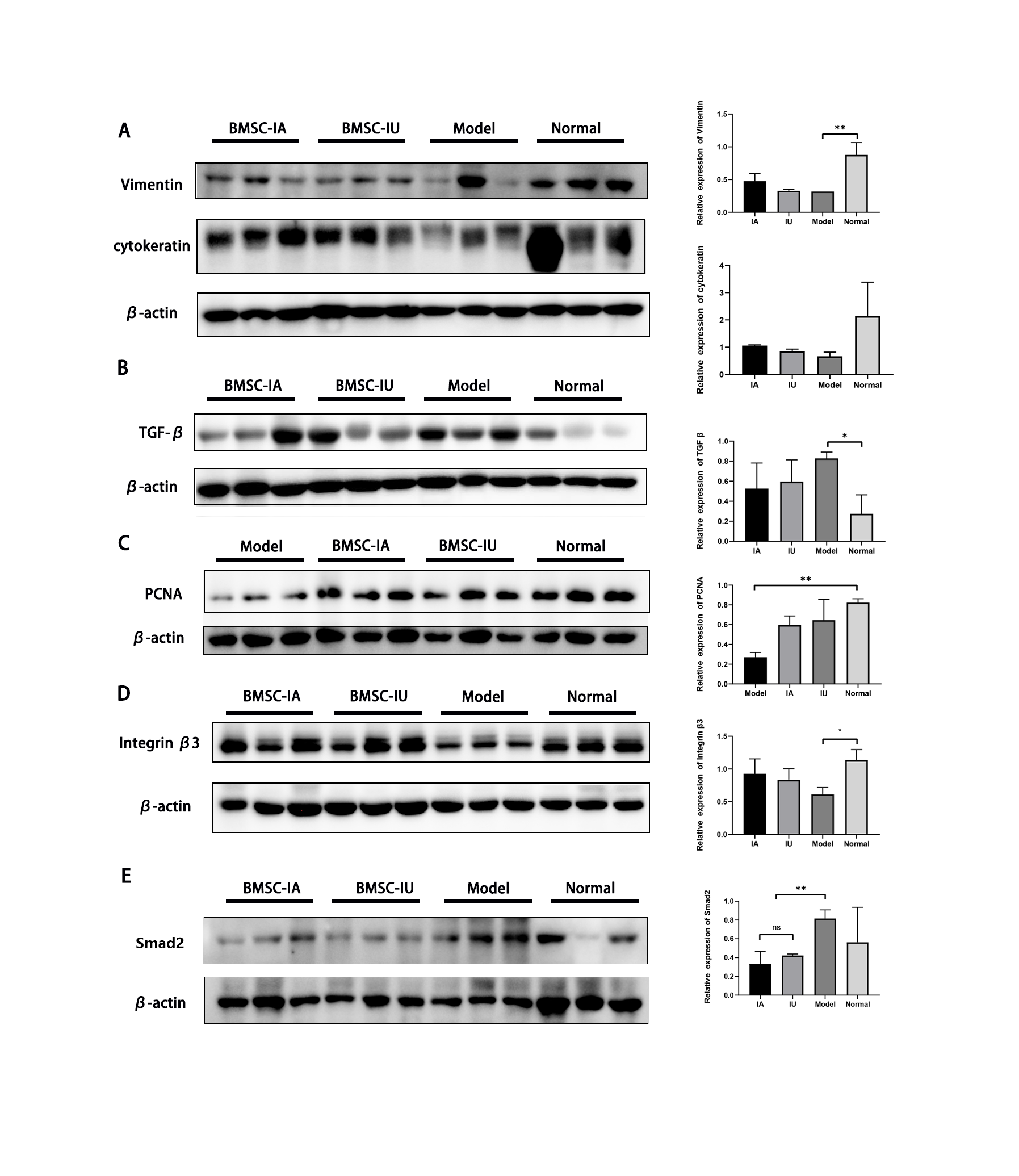

Supplement: Supplementary file 2 [file Image4.PNG]

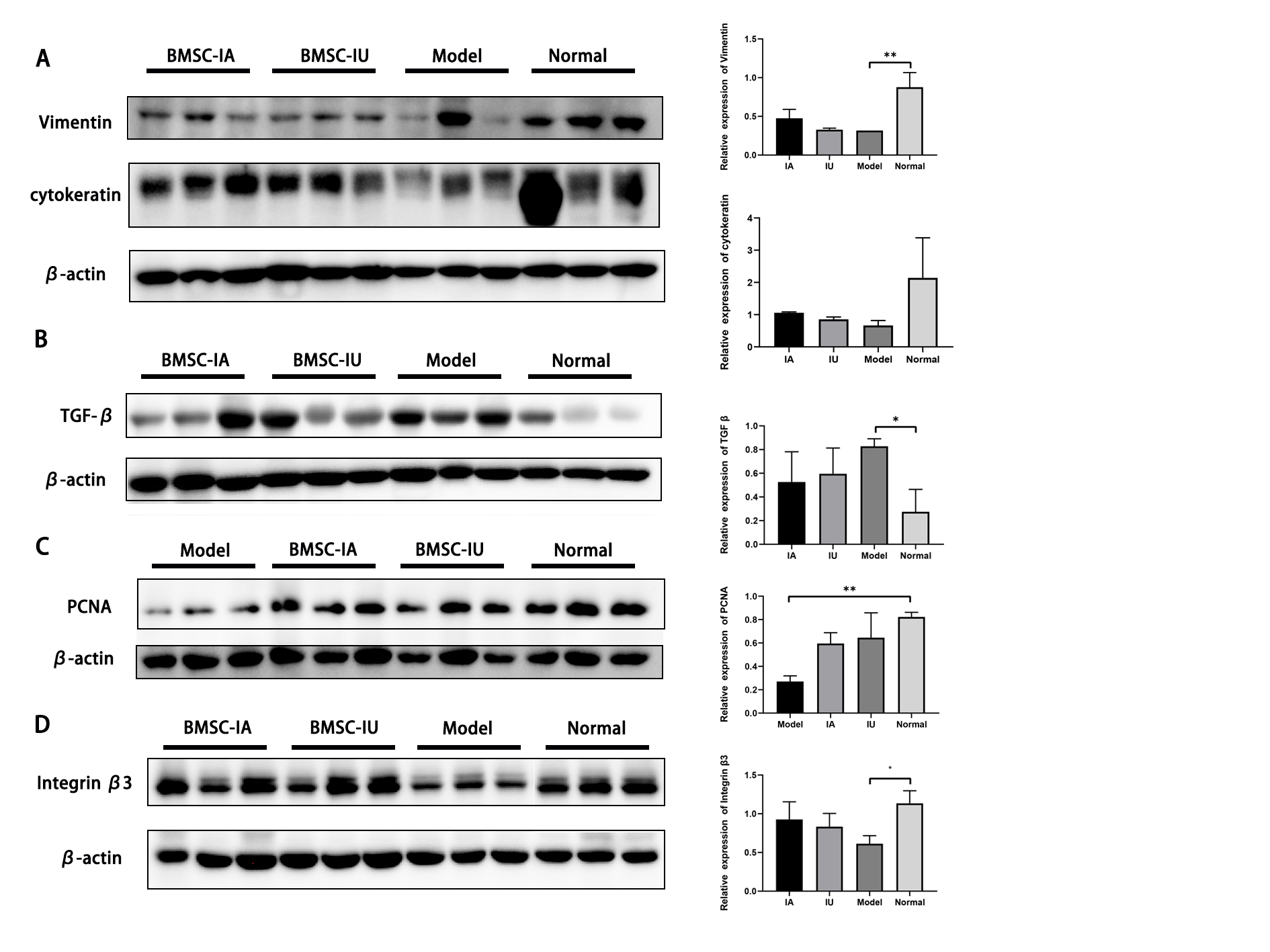

Supplement: Supplementary file 3 [file Image2.PNG]

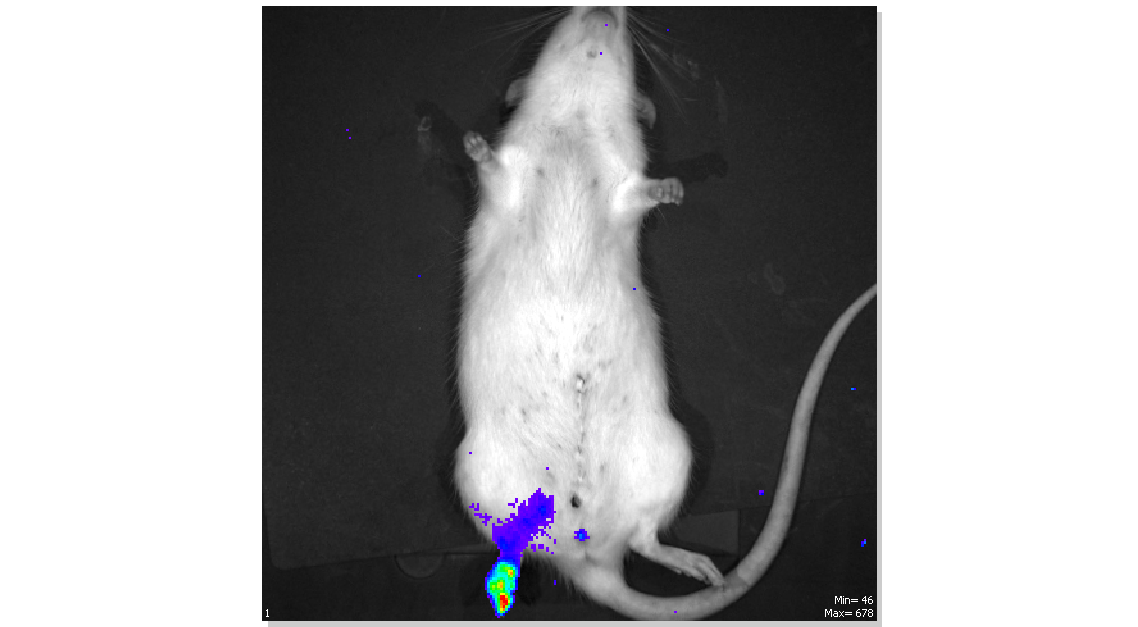

Supplement: Supplementary file 4 [file Image1.PNG]

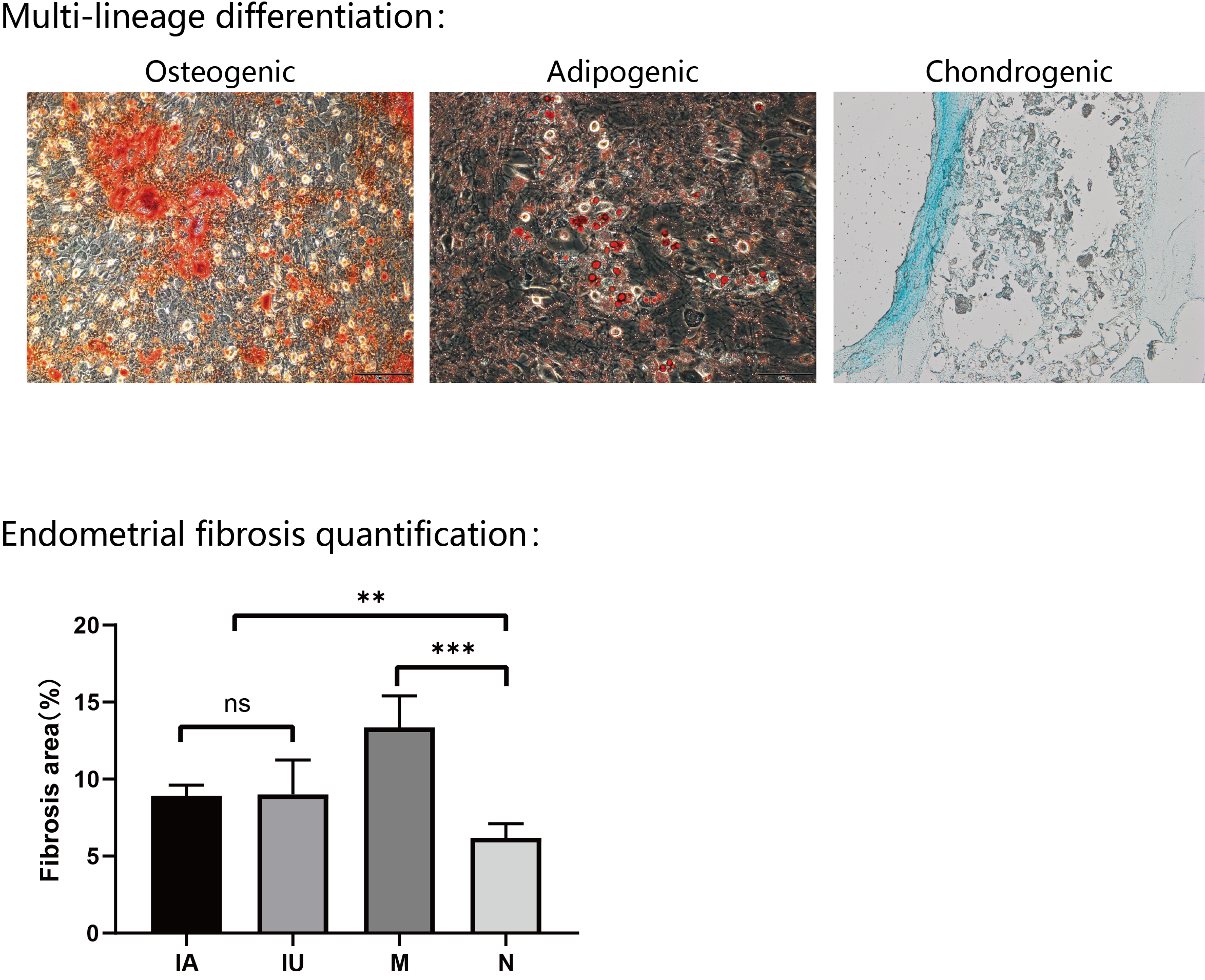

Supplement: Supplementary file 5 [file Image5.TIF]

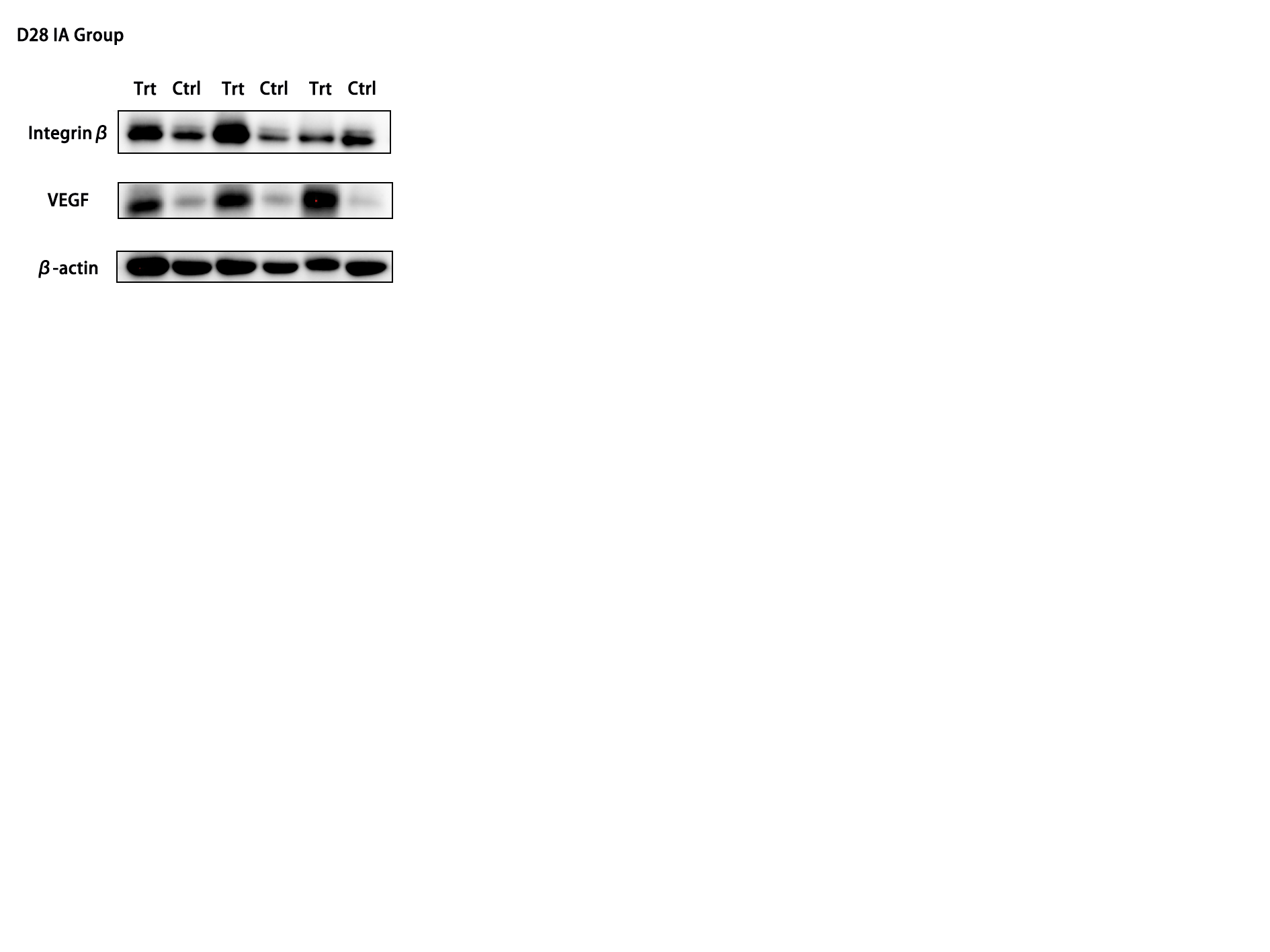

Supplement: Supplementary file 6 [file Image3.PNG]
